# DOACs – advances and limitations in real world

**DOI:** 10.1186/s12959-016-0111-3

**Published:** 2016-10-04

**Authors:** Lai Heng Lee

**Affiliations:** Department of Haematology, Singapore General Hospital, 20, College Road, Academia Level 3, Singapore, 169856 Singapore

**Keywords:** Oral anticoagulation, Atrial fibrillation, Stroke, Venous thromboembolism, Bleeding

## Abstract

The group of new oral anticoagulants or NOACs, now termed direct oral anticoagulants or DOACs, with their favourable results from large scale phase III clinical trials, represent a major advancement and expanded armamentarium in antithrombotic therapy. Dabigatran, rivaroxaban, apixaban and edoxaban are now in clinical routine use for prevention and treatment of arterial and venous thrombotic diseases as addressed in their clinical trials. Usage of the DOACs is expected to increase as clinicians gain more experience and reassurance with data from the real world studies which are generally consistent with that from clinical trials. Development of specific antidotes in management of bleeding complications and development of coagulation assays for their plasma levels will further boost the confidence in the DOACs. Nonetheless, there are still limitations associated with the DOACs. Many patients in need of anticoagulant therapy for indications not studied in the clinical trials will not be eligible for treatment with a DOAC. Conditions where more data is required include DOACs use in the paediatric age group, patients with atrial fibrillation and valvular heart disease, thrombosis associated with the anti-phospholipid syndrome and cancer associated thrombosis. The affordability and access to these drugs may pose an issue for many patients under healthcare systems not providing for these medications. With four new anticoagulants coming onboard very quickly, the focus has shifted to the practical approach and management in real life as many clinicians are not yet familiar with the DOACs. Clinicians need to be educated on how to manage this new class for drugs, from choosing the appropriate drug to prevention and managing bleeding complications as a lack of knowledge and understanding in these drugs will lead to inappropriate use and compromise on patient safety.

## Background

Oral anticoagulation is essential for treatment of arterial and venous thromboembolic diseases. Vitamin K antagonists such as warfarin and Coumadin were the only oral anticoagulant available for decades until the introduction of a group of new and novel oral anticoagulants, initially coined as NOACs. Their favourable pharmacological properties and convenient administration overcome many of the problems associated with the vitamin K antagonists such as frequent coagulation blood tests, dose adjustments and perceived dietary restrictions. In addition, the NOACs, as a class have been shown to have significantly less intracranial haemorrhage (ICH) than warfarin, thus mitigating the most feared complication of anticoagulation treatment. Subsequently, the term DOACs or ‘direct oral anticoagulants” was preferred to refer this class of oral anticoagulants with similar pharmacological properties that directly inhibit a single target [1]. Currently, four DOACs, namely Dabigatran, Rivaroxaban, Apixaban and Edoxaban are registered for use in prevention of stroke and systemic embolism in atrial fibrillation (AF), venous thromboembolism (VTE) prophylaxis in major orthopaedic surgery, treatment of acute VTE and prevention of recurrent VTE. In addition, Rivroxaban is also registered for use in prevention of cardiovascular deaths after acute coronary syndrome. With the DOACs becoming commercially available and more widely used, the focus has shifted to their practical use in real life. The purpose of this article is to highlight available phase IV or post marketing real world data and their consistency with their prior clinical trials and to highlight the limitations and challenges in using these agents in real world.

## Review

### Stroke prevention in non-valvular atrial fibrillation

Anticoagulation therapy is necessary to prevent stroke, systemic embolization and all-cause mortality in patients with AF. THE CHADS2 score (Cardiac Failure, Hypertension, Age, Diabetes, and Stroke [double]) was widely used in risk stratification to identify patients who will benefit from anticoagulation [2]. However, even within the score “0”, the risks of stroke ranged from 0.84 to 3.4 per year, hence missing out on those with increased risks who would have benefitted from anticoagulation. This gap was addressed with the CHA2DS2-VASc score (congestive heart failure, hypertension, age ≥ 75 years [doubled], diabetes mellitus, stroke [doubled], vascular disease, age 65–74 years, sex category [female]) which can better identify truly low risk AF patients, who are unlikely to benefit from antithrombotic therapy [3]. CHASDS2-VASc score is currently the preferred tool for risk stratification for stroke risk in AF patients.

Currently 4 DOACs available are dabigatran, rivaroxaban, apixaban and edoxaban have each shown similar efficacy and safety when compared to warfarin [4–7]. A meta-analysis of the phase III trials of these four DOACs showed a consistent favourable risk-benefit profile across a wide range of patients with significant reductions in stroke or systemic embolism, intracranial haemorrhage, and mortality but increased gastrointestinal bleeds when compared with warfarin [8]. Coupled with their convenient usage as fixed dose oral medications without the need for frequent laboratory tests and dose adjustments, DOACs have emerged as the preferred treatment option in some guidelines [9]. Large scale real world data of these drugs is has become available with increasing use of DOACs in routine care.

Dabigatran, in a review of 9 publications, involving more than 200,000 AF patients over 5 years [10], showed that data for dabigatran in ‘real world’ clinical practice were largely replicative of the main findings in the RE-LY phase III trial [4]. In particular, both dabigatran doses at 150 mg and 110 mg twice daily were associated with lower major extra-cranial bleeding rates than warfarin in patients less than75 years old and similar event rates in those above 75 years old. The 110 mg dose was associated with lower and similar gastrointestinal (GI) bleeding rates, and the 150 mg dose yielded similar and higher GI bleeding rates in patients less than 75 years and more than 75 years old, respectively. A more recent systematic review and meta-analysis studied 348 750 patients (56.65 % warfarin, 40.2 % dabigatran-150 mg and 3.2 % dabigatran-110 mg) in routine care and this is 20 times the size of RE-LY patient population [11]. It included heterogeneous study cohorts regarding history of stroke, hypertension, or diabetes mellitus and did not exclude patients with severe renal impairment (creatinine clearance ≤30 mL/min), active liver disease, or conditions associated with an increased risk of bleeding. Patients receiving dabigatran-110 mg in routine clinical practice, tended to be older than patients in the RE-LY trial on this dose. In pooled analyses, dabigatran-150 mg was similar to warfarin in preventing stroke (hazard ratio, 0.92; 95 % confidence interval, 0.84–1.01; *P* = 0.066) and a significantly lower risks of intracranial bleeding (0.44; 0.34–0.59; *P* < 0.001). However risks of GI bleeding was significantly higher than warfarin (1.23; 1.01–1.50; *P* = 0.041), particularly in studies of older versus younger populations (median/mean age, ≥75 versus <75 years; β = 1.53; 95 % confidence interval, 1.10–2.14; *P* = 0.020). Again the findings were consistent with the RE-LY trial. Another observational study provided some insights on the use of Dabigatran 75 mg dose which was not studied in the RE-LY trial but approved in the USA for use in the renal impaired patients with CrCl 15-30 ml/min [12]. The use of dabigatran 75 mg was associated with significantly reduced risk of intracranial haemorrhage and similar rates of stroke, bleeding and mortality compared to warfarin. Interestingly, majority of patients on dabigatran 75 mg twice daily appeared not to have severe renal impairment as only 33 % had a diagnosis of chronic kidney disease, and 20 % of these with severe renal impairment, thus suggesting a possible off-label use of the 75 mg dose in many patients in the real world. While not advocating off label dose reduction, the observational data are nonetheless reassuring. Other findings noted from such real world observations include the observations that new starters of warfarin has a higher bleeding risk when compared to new starters of dabigatran, warfarin experienced switchers or patients remaining on warfarin [13], higher bleeding rates in the first 90 days of treatment in elderly new starters of dabigatran or warfarin [12, 14] and the higher bleeding risk with renal impairment regardless of which oral anticoagulant [15–17].

Rivaroxaban, in the Xantus prospective observational study for 6784 AF patients across 311 centres in Europe reported a lower thrombotic and bleeding rates for Rivaroxaban compared to its Rocket phase III clinical trial [18]. While the phase III ROCKET AF trial did not include patients with CHADs score of 0-1, Xantus had 12.7 % of patients had a CHA2DS2-VASc score of either 0 or 1. Generally, patients in Xantus had lower stroke risks, with a mean CHADS2 score of 2.0 and 19.0 % experiencing prior stroke/TIA or SE, compared with 3.5 and 55 % respectively in the Rocket AF trial. The overall bleeding incidence of 2.1 per 100 patient-years in Xantus was notably lower than 3.6 per 100 patient-years reported in Rocket AF. Similarly fewer major GI bleeds and ICH were observed in Xantus when compared to Rocket-AF. Recent analysis from the Dresden Registry [19] showed the overall rates of stroke and systemic embolism at 2.03/100 patient-years in the intention-to-treat analysis and 1.7/100 patient-years in the on-treatment analysis which were considerably lower than those in the ROCKET AF trial [5]. In addition, event rates for patients receiving 20 mg OD (1.25/100 patient-years), was considerably lower than patients on 15 mg OD (2.7/100 patient-years). Bleeding complications associated with rivaroxaban was addressed in a meta-analysis of 9 studies involving 51,533 patients in real world [20]. It showed the mean pooled rates of any major bleeding, major GI bleeding or ICH with rivaroxaban were 3.32, (95 % CI¼2.28–4.25); 2.41, (95 % CI¼1.25–3.56) and 0.40, (95 % CI¼0.17–0.74) events/100 patient-years. The pooled real-world rates of these bleeding rates largely mirrored those reported for rivaroxaban in the phase 3 ROCKET AF trial [5]. However, there were significant variability and heterogeneity variability in major bleeding rates across the studies. Five studies were retrospective claims analyses and identified bleeding using International Classification of Diseases–9/10 codes, while four were prospective registry studies and identified bleeding clinically using the International Society on Thrombosis and Haemostasis (ISTH) definition. Major bleeding rates and major GI bleeding rates as per 100 patient-years in studies that relied on claims were (2.86 to 12.79) and (2.53 to 9.5) respectively and these are substantially higher than (0.96 to 3) and (0.19 to 0.9) as reported in prospective studies using clinical identification. Such differences underscore the substantial heterogeneity across the studies and the inherent weaknesses associated with retrospective and observational studies. Nonetheless, the pooled rates of major bleeding with rivaroxaban estimated were generally low and consistent with those reported in ROCKET AF. This finding of a lower major bleed rate than Rocket AF was also seen in a large US study of electronic medical records of 27 467 patients (2.9 events per 100 patient-years) [18] as well as an earlier report from Dresden NOAC Registry involving 1200 AF patients treated with rivaroxaban(3.1 events per 100 patient-years) [21, 22].

Besides these published large scale real world comparisons of a single DOAC versus warfarin, evidence relating to the overall effectiveness and safety of all oral anticoagulant drugs used in clinical practice is emerging. An observational nationwide cohort study in Denmark had involved 61, 678 patients with non-valvular AF who were naïve to oral anticoagulants and had no previous indication for valvular AF or VTE [23]. The study population was distributed according to treatment type with 57 % warfarin, 21 % dabigatran 150 mg, 20 % on rivaroxaban 20 mg, and 10 % apixaban 5 mg. The baseline characteristics of patients in apixaban and rivaroxaban has more previous strokes, systemic embolism vascular disease and bleeding while dabigatran patients were younger and less renal impaired, warfarin has more patients with vascular disease hypertension, renal impairment, COPD and cancer. During 1 year follow-up, when compared to warfarin, annual rates of ischaemia strokes and systemic embolism were significantly lower for rivaroxaban (hazard ratio 0.83 (95 % confidence interval 0.69 to 0.99), while not significantly different for dabigatran and apixaban.(hazard ratios of 2.8 % and 4.9 % respectively) The mortality risk was significantly lower with apixaban (5.2 %) and dabigatran (2.75 %) when compared with warfarin (8.5 %), but not with rivaroxaban (7.7 %). No significant difference was found between DOACs and warfarin for ischaemic stroke. The bleeding endpoints for rivaroxaban 5.3 % was comparable to warfarin 5 %, while apixaban 2.3 % and dabigatran 2.4 % were both lower than warfarin. The risks of death, any bleeding, or major bleeding were significantly lower for apixaban and dabigatran compared with warfarin. This real world study concluded that all three DOACs seem to be safe and effective alternatives to warfarin in a routine care setting.

Real-life studies have their inherent weaknesses such as non-controlled and heterogeneous patient groups, uncontrolled influence of non-compliance, other concomitant medications and co-morbidities. However, they provide a wealth of data and insight into how DOACs are used in the real world. Despite the reassuring real world data on use of DOACs in routine care, the benefits of DOACs are not applicable to all patients. As shown in a smaller scale study involving 468 patient with AF from the UZ Brussel Stroke Registry, it was found that less than half of real life patients are eligible for therapy with one of the DOACs [24]. Reasons for non-eligibility include concomitant use of antiplatelet agents with apixaban, impaired renal function in dabigatran, concomitant use of rifampicin and anti-fungal drugs and presence of valvular heart diseases. More data are also required for AF patients on DOACs undergoing cardioversion or ablation. There are ongoing trials addressing some of these issues and their results together with more real world data can add more clarity to these limitations.

### DOACs in Asia for AF patients

AF with its risks for ischaemic stroke is expected to pose a huge health burden in Asia. Although the DOACs are emerging as the preferred class of anticoagulant for stroke prevention in AF, there are concerns if the results of their global clinical trials are applicable to Asians. Subgroups analysis of more than 8000 Asian patients was performed in a meta-analysis of 5 pivotal phase III trials for the four available DOACs, namely RE-LY, ROCKET AF, J-ROCKET AF, ARISTOTLE, and ENGAGE AF-TIMI 48 [25]. The results showed greater benefits of DOACs in Asians with a greater reduction in stroke and systemic emboli when compared to non-Asians. As for bleeding complications, Asians also fared better with fewer bleeds than non-Asians. This is despite Asian patients on the warfarin comparator arm having less optimal time in therapeutic range, with more having international normalized ratio <2.0 and fewer had international normalized ratio >3.0. In particular, bleeding from the gastrointestinal track was similar between Asians on DOACs and vitamin K antagonists, but increased in non-Asians who were on DOACs. In studying the differences between Asians and non-Asians individually in each of the four DOACs [26], the relative risk reduction in stroke and systemic embolization, hemorrhagic stroke as well as all-cause mortality showed a greater numerical reduction in Asians compared to non-Asians for Dabigatran at 150 mg, 110 mg, Rivaroxaban 20 mg, Apixaban 5 mg and Edoxaban 60 mg. There was no evidence of increased risk of GI bleeding associated with DOACs in Asians. Hence it is expected that DOACs will become the preferred class of anticoagulant in the stroke prevention for AF in Asians.

In the real world, data the Taiwan National Health Insurance Research Database with close to 10,000 AF patients on each arm of treatment with either warfarin or dabigatran, has shown that Dabigatran significantly reduced risk of ischemic stroke, ICH, all hospitalized major bleeding and all-cause mortality compared with warfarin [27]. Rates of major GI bleeding and myocardial infarction were not increased in dabigatran when compared with warfarin. A multicenter retrospective cohort study of 241 stroke centers in Japan, patients with AF treated with a DOAC when compared with those on warfarin, had a lower rates of intracranial haemorrhage (17 % vs 26 %) and mortality (16 % vs 35 %) [28]. Such real world reports further bolster the confidence in the safety and efficacy of DOACs treatment for AF patients in Asia.

### Thrombo-prophylaxis in major orthopaedic surgeries

In the phase III clinical trials for thromboprophylaxis in major orthopaedic surgeries, rivaroxaban, dabigatran and apixaban were found to be effective and safe without a significant increase in bleeding complications when compared to enoxaparin 40 mg once daily [25] and edoxaban was found to be more effective when compared with enoxaparin 20 mg once daily [29–31]. With early discharge after surgery, the DOACs make a very attractive option for continued prophylaxis after hospital discharge.

In an observational study in routine practice using dabigatran for thrombo-prophylaxis in 5292 hip and knee replacement surgeries [32], for patients with pre-specified age, renal function and body mass index (BMI), the composite incidence of symptomatic VTE events and all-cause mortality was 1.04 % (95 % CI 0.78, 1.35) and the post hoc analysis incidence of major bleeding events to be consistent with the findings in clinical trials, thus providing reassurance with regards to efficacy and safety of its use in routine practice. A three-fold increase in symptomatic VTE and all-cause mortality was seen in patients with moderate renal impairment of creatinine clearance of 30–49/ml who received dabigatran and a 2 fold increase in major bleeding was seen in the severely obese patients with BMI above 35 kg/m2. High level of satisfaction with dabigatran use was also reported despite the difficult and time consuming process of implementing dabigatran into routine practice [33].

Rivaroxaban in routine care for thromboprophylaxis post major orthopaedic surgery was similarly evaluated. XAMOS, a phase IV, non-interventional study in 17,701 patients across 37 countries showed that the incidence of symptomatic thromboembolic events was significantly lower in patients who received rivaroxaban compared with standard of care (0.9 % vs 1.4 %) [34] Treatment related major bleeding events as defined in the RECORD programme were similar between the rivaroxaban and standard of care groups at 0.4 % and 0.3 %, respectively. However, non-major bleeding and any bleeding rates were higher in the rivaroxaban group compared with the standard of care group (2.9 % vs 1.7 %, and 4.7 % vs 3.2 %, respectively). Nonetheless the overall data from XAMOS confirmed the favourable benefit–risk profile of rivaroxaban when used in routine clinical care. From the ORTHO-TEP registry of about 5000 patients in a single centre, rivaroxaban vs fondaparinus or low molecular weight heparin was retrospectively evaluated for the prevention of VTE [35, 36]. The rivaroxaban group demonstrated a significant reduction in symptomatic VTE and a numerical reduction in pulmonary embolism. Significant reduction in major bleeding rates, number of surgical revisions due to bleeding complications, blood transfusion rates and length of hospital stay were also seen in the rivaroxaban group. Notwithstanding the inherent bias and inadequacies in retrospective study designs, ORTHO-TEP confirmed that the efficacy of rivaroxaban was at least not offset by any increase in surgical complications.

### Treatment of acute VTE

Similar to stroke prevention in patients with non-valvular AF, the DOACs have demonstrated excellent efficacy and safety in large scale phase III trials in the treatment of VTE [37–41]. Evidence from real life studies are pending from ongoing studies such as : (i) Dresden Noac Registry (clinicaltrials.gov identifier: NCT01588119), (ii) XALIA study which is a non-interventional observational cohort study investigating rivaroxaban in VTE treatment in routine clinical practice (clinicaltrials. gov identifier: NCT01619007), (iii) PREFER in VTE, a multicentre, prospective observational disease registry for quality of life and treatment satisfaction for 4000 patients with VTE across Europe [42] and (iv) The GARFIELD-VTE registry, an observational study for about 10,000 patients to look at the acute and long term management of VTE, its complications and healthcare resource utilization.

The use of DOACs in the treatment of VTE associated with Antiphospholipid requires more clarity as this condition is associated with much increased risks for recurrences. A prospective phase II/III clinical trial using Rivaroxaban in antiphospholipid syndrome is currently in progress to address these efficacy and safety issues [43]. Cancer associated VTE represents a good proportion of VTE patients for which low molecular weight treatment is regarded as the gold standard. While these patients are at higher risks of recurrence and bleeding, they may also be on medications which may interact with DOACs via the CYP3A4 and P-gp metabolic pathways. The main limitation in using DOACs is the lack of efficacy and safety data from clinical trials specific for use of DOACs in the treatment of cancer associated VTE. Although subgroup analysis of cancer patients from meta-analysis of the major clinical trials for DOACs in the treatment of VTE showed similar efficacy and safety when compared to warfarin, it is not clear if these cancer patients had active cancer, what were the risks of bleeding and recurrence specific to their cancer types and whether they are on cancer treatment with potential drug interaction with DOACs [44].

### Real life management issues

While it is reassuring that the body of emerging “real world data” from the routine use of NOACs largely mirrors that in clinical trials, there remains many concerns on the limitations of this new armamentarium for better management of thrombotic diseases.

### Appropriate choice of anticoagulant and patient care when using DOACs

Choosing a particular DOAC for a patient can be difficult as not all DOACs are the same [45]. There is no evidence to recommend one agent over another because the DOACs have never been compared in head-to-head trials. Hence the guiding principle has been to match the right drug to the right patient based on the dosing properties of each drug, the efficacy and safety and side effect profile as demonstrated in phase III trials and real world data, as well as the compliance, affordability and accessibility of the DOACs. Therefore understanding the properties of the drugs is fundamental to ensure safe and appropriate prescribing practice (Table 1). All DOACs depend variably on renal excretion; they are contraindicated in renal failure and should be used with caution with dose reductions in the renal impaired patients. The relative lack of drug interactions in DOACs is an advantage but clinicians must be mindful of the few important drug interactions with some anti-fungal, anti-microbial and anti-viral medications involving the CYP3A4 and P-gp metabolic pathways. The dosing regimen and dose adjustments for renal impairment are DOAC specific and different from each other and thus predispose to dosing and prescription errors if clinicians are not familiar with these anticoagulants. There are also clinical situations where DOACs are not suitable because of insufficient data on its efficacy and safety such as thromboembolism associated with anti-phospholipid syndrome and the unstable cancer patient, or where there are safety concerns as during pregnancy and for patient with mechanical heart valves. The notion of a simplified coagulation treatment approach is actually more complicated than anticipated and guidelines such as the NICE guidelines (https://www.nice.org.uk/guidance/cg180/resources/nic-consensus-statement-on-the-use-of-noacs-243733501) and the EHRA practical guide [46] are useful resources for clinicians prescribing DOACs. In situations when transiting between DOACs and other anticoagulants and during the peri-operative periods, due caution should be exercised taking into consideration the patient’s renal function and the half-life of the DOAC for appropriate drug dosing and time of administration. To minimise the bleeding risks, these agents should be used in the appropriate patients and managed well when transiting between DOAC and other anticoagulants and during the peri-operative periods [46].

**Table 1:** Pharmacological properties of the DOACs

	Dabigatran [9, 38]	Rivaroxaban [9, 40, 41]	Apixaban [9, 37]	Edoxaban [9, 39]
Target	Factor IIa	Factor Xa	Factor Xa	Factor Xa
Half-life (hour)	12-17	5-9	12	6-10
Time to peak effect (hour)	1-3	2-4	1-3	1-2
Renal clearance as unchanged drug (%)	80	33	27	50
Drug Interactions Pathways	P-gp	3A4/P-gp	3A4/P-gp	3A4/P-gp
Dosing in non-valvular AF	150 mg BID	20 mg OD	5 mg BID	60 mg OD
Dosing in VTE treatment	150 mg BID after 5-10 days of parenteral anticoagulation	15 mg BID for 21 days followed by 20 mg OD	10 mg BID for 7 days followed by 5 mg BID	60 mg OD after 5 days of parenteral anticoagulation

OD-once daily, BID-twice daily, P-gp – P-glycoprotein, 3A4 – cytochrome P450 3A4 isoenzyme

### Laboratory coagulation tests

Patients on DOACs do not need routine coagulation monitoring. However, there are certain clinical scenarios in which coagulation testing and measurement of drug levels are necessary, such as episodes of bleeding, perioperative management, suspected over dosage either from drug interactions or intentional overdose, renal impairment or a measure of suspected non-compliance. The effects of DOACs on routinely available clot-based tests such as APTT, PT and TT are variable and the degree of prolongation is highly dependent on the reagent used for the assay [47]. These widely available tests may be used to detect peak or supra-therapeutic drug levels, but should not be used for quantitation. Such routine coagulation tests may also appear normal during trough drug levels. In situations where drug levels are required and these are measured with specialized tests [48]. Dabigatran can be measured by the dilute thrombin time, ecarin assay and chromogenic anti-IIa assay. The anti-Xa DOACs can be measured by STA Neoplastin test and the anti-Xa assays. Unfortunately, such specialised laboratory facilities are not widely available, except in major hospitals, and very often not available when required.

In addition to influencing the results of clotting times, DOACs may also cause potential errors in some specialized coagulation tests, particularly, the clot based assays [49]. DOACs can falsely prolong the dilute Russels Vipers Venom Time (dRVVT) in lupus anticoagulant tests, falsely reduced levels of plasma clotting factors in one staged assays and falsely elevate the functional protein S levels. Generally, clinicians may not appreciate the impact of DOACs on coagulation tests, hence guidance from the local laboratory is important for appropriate requests, timing and interpretation of these tests for patients on DOAC treatment [50].

### Management of DOAC associated bleeding

All anticoagulants, including the DOACs are associated with bleeding. Unlike warfarin and heparins where established reversal protocols with known antidotes are readily available for managing bleeds, clinicians may not be equipped with the bleeding management and reversal strategies in DOACs [51]. It is imperative that clinicians be equipped to manage bleeds by means of a local management plan stratified according to the severity of bleeds and availability of treatment agents [52] Exact identification of which DOAC, location and severity of bleeds, renal function and the time of last ingestion of DOAC are important factors to note in the management of bleeding associated with DOACs.

For mild bleeding events such as bruises or menorrhagia, the DOAC could be suspended and the drug restarted later at a lower dose and resuming full dose when bleed risks had resolved. For moderate to severe bleeds, symptomatic treatment such as mechanical compression of bleeding site, appropriate surgical or radiological intervention and blood transfusion may be necessary. Plasma and cryoprecipitate do not reverse the anticoagulant effect of novel agents, but may be required for correction of volume loss or coagulopathy associated with other co-morbidities. Adsorption of remnant DOACs by oral activated charcoal may be helpful if the last ingested dose of is within 2 h at presentation to the hospital [53]. Haemodialysis, with its logistic challenges, can be considered for removal of residual dabigatran [54] but not for the anti-Xa inhibitors like rivaroxaban or apixaban.

In the events of life threatening bleeds, off label use of pharmacological haemostatic agents such as prothrombin concentration complex and recombinant factor VIIa can be considered. However, such measures are controversial and without good supportive evidence [55]. Clinical development for specific antidotes to the DOACs took place only after DOACs reached routine use. The humanized monoclonal Fab BI 655075 or Idarucizumab specific for reversal of the anticoagulant effects of dabigatran has completed its phase III trial [56]. It has now been registered for use in many countries. Andexanet alfa [57], a FXa inhibitor antidote, is in advanced stages of clinical trial development. It is a recombinant modified human factor Xa decoy protein that is catalytically inactive but binds factor Xa inhibitors in the active site with high affinity, hence restoring the activity of endogenous factor Xa and reducing levels of anticoagulant activity. The availability of specific antidotes for the DOACs will certainly provide a better sense of security into their routine use. However, it is prudent to highlight the appropriate use of such antidotes [58]. Reasonable indications include life threatening and critical organ bleeds, bleeding that did not respond to conventional supportive and hemostatic measures, and for urgent surgical and invasive interventions with high risks of bleed that cannot be delayed. In the decision to reverse anticoagulation completely, one must take into consideration the pro-thrombotic risks that the patient has which required anticoagulation in the first place, hence such reversal should be taken lightly for trivial reasons just because reversal agents are available.

### Cost effectiveness of DOACs

Affordability and accessibility for DOACs remains an issue in Asia. While some studies have advocated the positive cost savings and cost-effectiveness of DOACs in routine clinical practices [59–61], these may not be applicable to many countries with different healthcare systems. Also, the cost effectiveness as calculated from healthcare providers’ perspective may not be portable to the full paying individual without subsidized healthcare benefits [62].

## Conclusion

DOACs represent an important advancement in antithrombotic management. Real world data consistent with their clinical trial data is reassuring. However, a significant number of patients may not be eligible for treatment with a DOAC from a lack of data in conditions not addressed in their clinical trials. In addition, costs of these medications may limit their accessibility to a good number of patients. In routine clinical care, much has to be done to familiarize the clinicians on the use of these drugs. It is the careful patient selection, appropriate management and the ability to prevent and manage the bleeding complications that will permit optimization on the use of DOACs.

## Abbreviations

AF: Atrial fibrillation
DOACS: Direct oral anticoagulants
GT: Gastrointestinal
ICH: Intracranial haemorrhage.
NOACs: New oral anticoagulants
VTE: Venous thromboembolism

## References

[1] Barnes GD, Ageno W, Ansell J, Kaatz S (2015). Recommendation on the nomenclature for oral anticoagulants: communication from the SSC of the ISTH. J Thromb Haemost.

[2] Keogh C, Wallace E, Dillon C, Dimitrov BD, Fahey T (2011). Validation of the CHADS2 clinical prediction rule to predict ischaemic stroke. A systematic review and meta-analysis. Thromb Haemost.

[3] Olesen JB, Torp-Pedersen C, Hansen ML, Lip GY (2012). The value of the CHA2DS2-VASc score for refining stroke risk stratification in patients with atrial fibrillation with a CHADS2 score 0-1: a nationwide cohort study. Thromb Haemost.

[4] Connolly SJ, Ezekowitz MD, Yusuf S, Eikelboom J, Oldgren J, Parekh A (2009). Dabigatran versus warfarin in patients with atrial fibrillation. N Engl J Med.

[5] Patel MR, Mahaffey KW, Garg J, Pan G, Singer DE, Hacke W (2011). Rivaroxaban versus warfarin in nonvalvular atrial fibrillation. N Engl J Med.

[6] Granger CB, Alexander JH, McMurray JJ, Lopes RD, Hylek EM, Hanna M (2011). Apixaban versus warfarin in patients with atrial fibrillation. N Engl J Med.

[7] Giugliano RP, Ruff CT, Braunwald E, Murphy SA, Wiviott SD, Halperin JL (2013). Edoxaban versus warfarin in patients with atrial fibrillation. N Engl J Med.

[8] Ruff CT, Giugliano RP, Braunwald E, Hoffman EB, Deenadayalu N, Ezekowitz MD (2014). Comparison of the efficacy and safety of new oral anticoagulants with warfarin in patients with atrial fibrillation: a meta-analysis of randomised trials. Lancet.

[9] Camm AJ, Lip GY, De Caterina R, Savelieva I, Atar D, Hohnloser SH (2012). 2012 focused update of the ESC Guidelines for the management of atrial fibrillation: an update of the 2010 ESC Guidelines for the management of atrial fibrillation--developed with the special contribution of the European Heart Rhythm Association. Europace.

[10] Potpara TS (2015). Dabigatran in ‘real-world’ clinical practice for stroke prevention in patients with non-valvular atrial fibrillation. Thromb Haemost.

[11] Romanelli RJ, Nolting L, Dolginsky M, Kym E, Orrico KB (2016). Dabigatran versus warfarin for atrial fibrillation in real-world clinical practice: A systematic review and meta-analysis. Circ Cardiovasc Qual Outcomes.

[12] Graham DJ, Reichman ME, Wernecke M, Zhang R, Southworth MR, Levenson M (2015). Cardiovascular, bleeding, and mortality risks in elderly Medicare patients treated with dabigatran or warfarin for nonvalvular atrial fibrillation. Circulation.

[13] Larsen TB, Gorst-Rasmussen A, Rasmussen LH, Skjoth F, Rosenzweig M, Lip GY (2014). Bleeding events among new starters and switchers to dabigatran compared with warfarin in atrial fibrillation. Am J Med.

[14] Avgil-Tsadok M, Jackevicius CA, Essebag V, Eisenberg MJ, Rahme E, Behlouli H (2016). Dabigatran use in elderly patients with atrial fibrillation. Thromb Haemost.

[15] Hernandez I, Baik SH, Pinera A, Zhang Y (2015). Risk of bleeding with dabigatran in atrial fibrillation. JAMA Intern Med.

[16] Lauffenburger JC, Farley JF, Gehi AK, Rhoney DH, Brookhart MA, Fang G (2015). Effectiveness and safety of dabigatran and warfarin in real-world US patients with non-valvular atrial fibrillation: a retrospective cohort study. J Am Heart Assoc.

[17] Seeger JD, Bykov K, Bartels DB, Huybrechts K, Zint K, Schneeweiss S (2015). Safety and effectiveness of dabigatran and warfarin in routine care of patients with atrial fibrillation. Thromb Haemost.

[18] Camm AJ, Amarenco P, Haas S, Hess S, Kirchhof P, Kuhls S (2016). XANTUS: a real-world, prospective, observational study of patients treated with rivaroxaban for stroke prevention in atrial fibrillation. Eur Heart J.

[19] Hecker J, Marten S, Keller L, Helmert S, Michalski F, Werth S (2016). Effectiveness and safety of rivaroxaban therapy in daily-care patients with atrial fibrillation. Results from the Dresden NOAC Registry. Thromb Haemost.

[20] Weeda ER, White CM, Peacock WF, Coleman CI (2016). Rates of major bleeding with rivaroxaban in real-world studies of nonvalvular atrial fibrillation patients: a meta-analysis. Curr Med Res Opin.

[21] Tamayo S, Frank Peacock W, Patel M, Sicignano N, Hopf KP, Fields LE (2015). Characterizing major bleeding in patients with nonvalvular atrial fibrillation: a pharmacovigilance study of 27 467 patients taking rivaroxaban. Clin Cardiol.

[22] Beyer-Westendorf J, Forster K, Pannach S, Ebertz F, Gelbricht V, Thieme C (2014). Rates, management, and outcome of rivaroxaban bleeding in daily care: results from the Dresden NOAC registry. Blood.

[23] Larsen TB, Skjoth F, Nielsen PB, Kjaeldgaard JN, Lip GY (2016). Comparative effectiveness and safety of non-vitamin K antagonist oral anticoagulants and warfarin in patients with atrial fibrillation: propensity weighted nationwide cohort study. BMJ.

[24] Desmaele S, Steurbaut S, Cornu P, Brouns R, Dupont AG (2016). Clinical trials with direct oral anticoagulants for stroke prevention in atrial fibrillation: how representative are they for real life patients?. Eur J Clin Pharmacol.

[25] Wang KL, Lip GY, Lin SJ, Chiang CE (2015). Non-vitamin K antagonist oral anticoagulants for stroke prevention in Asian patients with nonvalvular atrial fibrillation: meta-analysis. Stroke.

[26] Chiang CE, Wang KL, Lin SJ (2015). Asian strategy for stroke prevention in atrial fibrillation. Europace.

[27] Chan YH, Yen KC, See LC, Chang SH, Wu LS, Lee HF (2016). Cardiovascular, bleeding, and mortality risks of dabigatran in Asians with nonvalvular atrial fibrillation. Stroke.

[28] Saji N, Kimura K, Aoki J, Uemura J, Sakamoto Y (2015). Intracranial hemorrhage caused by non-vitamin K antagonist oral anticoagulants (NOACs)-multicenter retrospective cohort study in Japan. Circ J.

[29] Adam SS, McDuffie JR, Lachiewicz PF, Ortel TL, Williams JW (2013). Comparative effectiveness of new oral anticoagulants and standard thromboprophylaxis in patients having total hip or knee replacement: a systematic review. Ann Intern Med.

[30] Fuji T, Wang CJ, Fujita S, Kawai Y, Kimura T, Tachibana S (2014). Safety and efficacy of edoxaban, an oral factor xa inhibitor, for thromboprophylaxis after total hip arthroplasty in Japan and Taiwan. J Arthroplasty.

[31] Fuji T, Wang CJ, Fujita S, Kawai Y, Nakamura M, Kimura T (2014). Safety and efficacy of edoxaban, an oral factor Xa inhibitor, versus enoxaparin for thromboprophylaxis after total knee arthroplasty: the STARS E-3 trial. Thromb Res.

[32] Rosencher N, Samama CM, Feuring M, Brueckmann M, Kleine E, Clemens A (2016). Dabigatran etexilate for thromboprophylaxis in over 5000 hip or knee replacement patients in a real-world clinical setting. Thromb J.

[33] Kendoff D, Perka C, Fritsche HM, Gehrke T, Hube R (2011). Oral thromboprophylaxis following total hip or knee replacement: review and multicentre experience with dabigatran etexilate. Open Orthop J.

[34] Turpie AG, Haas S, Kreutz R, Mantovani LG, Pattanayak CW, Holberg G, Jamal W (2014). A non-interventional comparison of rivaroxaban with standard of care for thromboprophylaxis after major orthopaedic surgery in 17,701 patients with propensity score adjustment. Thromb Haemost.

[35] Beyer-Westendorf J, Lutzner J, Donath L, Radke OC, Kuhlisch E, Hartmann A (2012). Efficacy and safety of rivaroxaban or fondaparinux thromboprophylaxis in major orthopedic surgery: findings from the ORTHO-TEP registry. J Thromb Haemost.

[36] Beyer-Westendorf J, Lutzner J, Donath L, Tittl L, Knoth H, Radke OC (2013). Efficacy and safety of thromboprophylaxis with low-molecular-weight heparin or rivaroxaban in hip and knee replacement surgery: findings from the ORTHO-TEP registry. Thromb Haemost.

[37] Agnelli G, Buller HR, Cohen A, Curto M, Gallus AS, Johnson M (2013). Oral apixaban for the treatment of acute venous thromboembolism. N Engl J Med.

[38] Schulman S, Kakkar AK, Goldhaber SZ, Schellong S, Eriksson H, Mismetti P (2014). Treatment of acute venous thromboembolism with dabigatran or warfarin and pooled analysis. Circulation.

[39] Buller HR, Decousus H, Grosso MA, Mercuri M, Middeldorp S, Prins MH (2013). Edoxaban versus warfarin for the treatment of symptomatic venous thromboembolism. N Engl J Med.

[40] Bauersachs R, Berkowitz SD, Brenner B, Buller HR, Decousus H, Gallus AS (2010). Oral rivaroxaban for symptomatic venous thromboembolism. N Engl J Med.

[41] Buller HR, Prins MH, Lensin AW, Decousus H, Jacobson BF, Minar E (2012). Oral rivaroxaban for the treatment of symptomatic pulmonary embolism. N Engl J Med.

[42] Agnelli G, Gitt AK, Bauersachs R, Fronk EM, Laeis P, Mismetti P (2015). The management of acute venous thromboembolism in clinical practice - study rationale and protocol of the European PREFER in VTE Registry. Thromb J.

[43] Cohen H, Dore CJ, Clawson S, Hunt BJ, Isenberg D, Khamashta M (2015). Rivaroxaban in antiphospholipid syndrome (RAPS) protocol: a prospective, randomized controlled phase II/III clinical trial of rivaroxaban versus warfarin in patients with thrombotic antiphospholipid syndrome, with or without SLE. Lupus.

[44] van der Hulle T, den Exter PL, Kooiman J, van der Hoeven JJ, Huisman MV, Klok FA (2014). Meta-analysis of the efficacy and safety of new oral anticoagulants in patients with cancer-associated acute venous thromboembolism. J Thromb Haemost.

[45] Schaefer JK, McBane RD, Wysokinski WE (2016). How to choose appropriate direct oral anticoagulant for patient with nonvalvular atrial fibrillation. Ann Hematol.

[46] Heidbuchel H, Verhamme P, Alings M, Antz M, Diener HC, Hacke W (2015). Updated European Heart Rhythm Association Practical Guide on the use of non-vitamin K antagonist anticoagulants in patients with non-valvular atrial fibrillation. Europace.

[47] Tripodi A (2013). The laboratory and the direct oral anticoagulants. Blood.

[48] Dale BJ, Chan NC, Eikelboom JW (2016). Laboratory measurement of the direct oral anticoagulants. Br J Haematol.

[49] Tsakiris DA (2014). Direct oral anticoagulants--interference with laboratory tests and mechanism of action. Semin Hematol.

[50] Wong WH, Yip CY, Sum CL, Tan CW, Lee LH, Yap ES (2016). A practical guide to ordering and interpreting coagulation tests for patients on direct oral anticoagulants in Singapore. Ann Acad Med Singapore.

[51] Siegal DM, Garcia DA, Crowther MA (2014). How I treat target-specific oral anticoagulant-associated bleeding. Blood.

[52] Ng HJ, Chee YL, Ponnudurai K, Lim LC, Tan D, Tay JC (2013). Consensus recommendations for preventing and managing bleeding complications associated with novel oral anticoagulants in singapore. Ann Acad Med Singapore.

[53] Wang X, Mondal S, Wang J, Tirucherai G, Zhang D, Boyd RA (2014). Effect of activated charcoal on apixaban pharmacokinetics in healthy subjects. Am J Cardiovasc Drugs.

[54] Khadzhynov D, Wagner F, Formella S, Wiegert E, Moschetti V, Slowinski T (2013). Effective elimination of dabigatran by haemodialysis. A phase I single-centre study in patients with end-stage renal disease. Thromb Haemost.

[55] Cuker A, Siegal D (2015). Monitoring and reversal of direct oral anticoagulants. Hematology Am Soc Hematol Educ Program.

[56] Pollack CV, Reilly PA, Eikelboom J, Glund S, Verhamme P, Bernstein RA (2015). Idarucizumab for Dabigatran Reversal. N Engl J Med.

[57] Siegal DM, Curnutte JT, Connolly SJ, Lu G, Conley PB, Wiens BL (2015). Andexanet Alfa for the reversal of Factor Xa inhibitor activity. N Engl J Med.

[58] Levy JH, Ageno W, Chan NC, Crowther M, Verhamme P, Weitz JI (2016). When and how to use antidotes for the reversal of direct oral anticoagulants: guidance from the SSC of the ISTH. J Thromb Haemost.

[59] Amin A, Bruno A, Trocio J, Lin J, Lingohr-Smith M (2016). Real-world medical cost avoidance when new oral anticoagulants are used versus warfarin for venous thromboembolism in the United States. Clin Appl Thromb Hemost.

[60] Jugrin AV, Ustyugova A, Urbich M, Lamotte M, Sunderland T (2015). The cost-utility of dabigatran etexilate compared with warfarin in treatment and extended anticoagulation of acute VTE in the UK. Thromb Haemost.

[61] Amin A, Stokes M, Makenbaeva D, Wiederkehr D, Wu N, Lawrence JH (2014). Estimated medical cost reductions associated with use of novel oral anticoagulants vs warfarin in a real-world non-valvular atrial fibrillation patient population. J Med Econ.

[62] Wang Y, Xie F, Kong MC, Lee LH, Ng HJ, Ko Y (2014). Cost-effectiveness of dabigatran and rivaroxaban compared with warfarin for stroke prevention in patients with atrial fibrillation. Cardiovasc Drugs Ther.

